# Development of a low-cost robotized 3D-prototype for automated optical microscopy diagnosis: An open-source system

**DOI:** 10.1371/journal.pone.0304085

**Published:** 2024-06-21

**Authors:** Allisson Dantas de Oliveira, Carles Rubio Maturana, Francesc Zarzuela Serrat, Bruno Motta Carvalho, Elena Sulleiro, Clara Prats, Anna Veiga, Mercedes Bosch, Javier Zulueta, Alberto Abelló, Elisa Sayrol, Joan Joseph-Munné, Daniel López-Codina

**Affiliations:** 1 Computational Biology and Complex Systems Group, Physics Department, Universitat Politècnica de Catalunya (UPC), Castelldefels, Spain; 2 Microbiology Department, Vall d’Hebron Research Institute (VHIR), Vall d’Hebron University Hospital, Barcelona, Spain; 3 Department of Microbiology and Genetics, Universitat Autònoma de Barcelona (UAB), Barcelona, Spain; 4 Department of Informatics and Applied Mathematics, Federal University of Rio Grande do Norte, Natal, Brazil; 5 CIBERINFEC, ISCIII- CIBER de Enfermedades Infecciosas, Instituto de Salud Carlos III, Madrid, Spain; 6 Probitas Foundation, Barcelona, Spain; 7 Database Technologies and Information Group, Service and Information Systems Engineering Department, Universitat Politècnica de Catalunya (UPC), Barcelona, Spain; 8 Tecnocampus, Universitat Pompeu Fabra, Mataró, Spain; University of Porto Faculty of Engineering: Universidade do Porto Faculdade de Engenharia, PORTUGAL

## Abstract

In a clinical context, conventional optical microscopy is commonly used for the visualization of biological samples for diagnosis. However, the availability of molecular techniques and rapid diagnostic tests are reducing the use of conventional microscopy, and consequently the number of experienced professionals starts to decrease. Moreover, the continuous visualization during long periods of time through an optical microscope could affect the final diagnosis results due to induced human errors and fatigue. Therefore, microscopy automation is a challenge to be achieved and address this problem. The aim of the study is to develop a low-cost automated system for the visualization of microbiological/parasitological samples by using a conventional optical microscope, and specially designed for its implementation in resource-poor settings laboratories. A 3D-prototype to automate the majority of conventional optical microscopes was designed. Pieces were built with 3D-printing technology and polylactic acid biodegradable material with Tinkercad/Ultimaker Cura 5.1 slicing softwares. The system’s components were divided into three subgroups: microscope stage pieces, storage/autofocus-pieces, and smartphone pieces. The prototype is based on servo motors, controlled by Arduino open-source electronic platform, to emulate the X-Y and auto-focus (Z) movements of the microscope. An average time of 27.00 ± 2.58 seconds is required to auto-focus a single FoV. Auto-focus evaluation demonstrates a mean average maximum Laplacian value of 11.83 with tested images. The whole automation process is controlled by a smartphone device, which is responsible for acquiring images for further diagnosis via convolutional neural networks. The prototype is specially designed for resource-poor settings, where microscopy diagnosis is still a routine process. The coalescence between convolutional neural network predictive models and the automation of the movements of a conventional optical microscope confer the system a wide range of image-based diagnosis applications. The accessibility of the system could help improve diagnostics and provide new tools to laboratories worldwide.

## Introduction

Microscopy is a fundamental pillar in laboratories all over the world. The observation of the forms by magnifying the image through microscope lenses facilitated laboratory practice. One of the main functionalities of microscopy is diagnostics. Conventional microscopy is still used globally in clinical laboratories as a standardized diagnostic technique. Moreover, it is still the *gold standard* technique for the diagnosis of many diseases, such as malaria and some Neglected Tropical Diseases (NTD) [1]. In addition, microscopy remains a crucial tool in resource-poor settings, where the availability of novel and more expensive techniques is limited. The accessibility and easy-handling of microscopy allows its usage in laboratories worldwide. The direct observation of biological samples is expert dependent; needs high levels of expertise and knowledge for each type of observed sample; and could trigger on diagnostic errors due to fatigue or long periods of visualization time [2]. This casuistry is producing a decrease in the use of traditional microscopy as a reference diagnostic method, although it is still the mainstay of laboratories worldwide. In addition, because of the increasing failure of malaria rapid diagnostic tests (RDTs) due to *pfhrp2/3* gene deletions [3] and the low sensitivity in cases of low parasite density [4], microcopy remains the gold standard and reference technique for malaria diagnosis. Furthermore, its relevance in the diagnosis of parasitic infectious diseases is still important, and should be considered as a reference method regardless of the novel molecular techniques and the loss of microscopy professionals.

Last years, novel technologies based on the automation of the microscope movements and image acquisition and processing are being developed to solve human fatigue and errors due to the continuous observation through the lenses. On the one hand, the complete robotization of a conventional optical microscope should improve the traditional microscopy in terms of autonomous diagnostics. For this purpose, servo motors can be a proper alternative to emulate microscope stage movements through the X-Y and Z (focus) axis, with 3D-printed pieces providing the mechanical support for the device to carry out this type of movement. On the other hand, several studies based on automatized image-based diagnosis with artificial intelligence (AI) models have been published during the last years, making use of Convolutional Neural Networks (CNNs) to automatically detect bacteria, cells, parasites or microalgae in digital images, thus, becoming a new alternative for traditional microscopy [5–7]. Interestingly, the combination of an autonomous microscope for image acquisition and an autonomous diagnosis by CNN image analysis might be the best solution for supporting microscopy-based diagnoses. The coalescence of both technologies generates a completely automated diagnostic procedure, from the movements of the microscope to the final diagnosis by image analysis. Moreover, low-cost systems are crucial for the easy and accessible implementation of automated microscopes in any laboratory worldwide, especially in endemic NTD regions and resource-poor settings [8].

As for the automation of a microscope for image acquisition, stage movements and auto-focus issue are the main strains. Ze-Jun 2012, developed an automatic movement stage with a parallelogram linkage mechanism in an optical microscope [9]. Sanz et al. 2021 reviewed state-of-the-art autofocus procedures in order to propose a general applicable methodology [10]. Some studies demonstrate, with promising results, the possibility to create that type of autonomous devices for the detection of diatoms [11], such as Salido et al. 2020, that designed a fully operative low-cost automated microscope for diatom detection with *you only look once* (YOLO) deep learning library [11]. Other applications for the autonomous detection of *Plasmodium* parasites in blood samples [12], or *Schistosoma haematobium* eggs in urine samples [13], were also postulated as alternative methods for diagnosis. Moreover, Alexandrov et al. 2020 designed a super-resolution high-speed optical microscope for automated readout of metallic nanoparticles and nanostructures [14]. As reviewed, microscope automation has a wide range of applications, mainly for the detection and observation of microscopic objects by the emulation of traditional microscopy.

In this study, we address the automation of a conventional optical microscope up to the image acquisition, as a first step to prepare it for a further automated diagnosis. We designed a low-cost 3D-prototype that can be adapted and implemented to several conventional optical microscopes. The prototype pieces were designed and built with 3D-printing technology. Microscope stage and focus movements were performed by servo motors guided by an Arduino controller. The whole procedure was monitored by a smartphone device, which was responsible for the robotized microscope movements and the acquisition of digital images for further analysis. The automated prototype was able to move the sample on the stage of the microscope through the X-Y axis (horizontal and vertical movements), and on the Z axis by an auto-focus algorithm. Its characteristics make the prototype available for any laboratory regardless of their resources or limitations. Importantly, this automated diagnostic system was designed to be open-source and available.

## Materials and methods

The robotized prototype for conventional optical microscopes was designed following the methods explained in the subsequent section.

### List of materials and devices

All material and devices specifications are listed in S1 Table. Below we present the list of materials and devices employed for the design, development and manufacturing of the robotized system.

- A conventional optical microscope with the standard characteristics to perform a diagnosis [15]. Binocular, with quadruple revolving objectives of 4x, 10x, 40x and 100x (immersion oil) magnification; and an ocular lens of 10x magnification. The microscope should consist of a fine and coarse adjustment wheels to focus the sample through the Z-axis, a microscope stage to deposit the sample with motion along X-Y axes, and light illumination.
- A 3D-printer Ender-3 Pro (Creality 3D). Ultimaker Cura 5.1 slicing software [16] and Tinkercad open-source software were used to design and print the 3D pieces. The filament employed for 3D pieces manufacturing was a Polylactic Acid (PLA), an easy-to-use, low-cost, biodegradable, and recyclable material.
- Three Micro Servo Motors 9G. The servo motors were employed to move the sample on the microscope stage through the X-Y-Z axes.
- Arduino MKR WiFi 1010 based on SAMD21 Cortex®-M0+ 32bit low power ARM® MCU microcontroller.
- Electronic components: cables, LED lights, and resistances (200 Ω).
- For the development of the autonomous microscope, three models of smartphones have been operated. A Samsung Galaxy S20, a Xiaomi Redmi 10C and a Samsung Galaxy A13.

### Robotized system design

**Fig 1:**
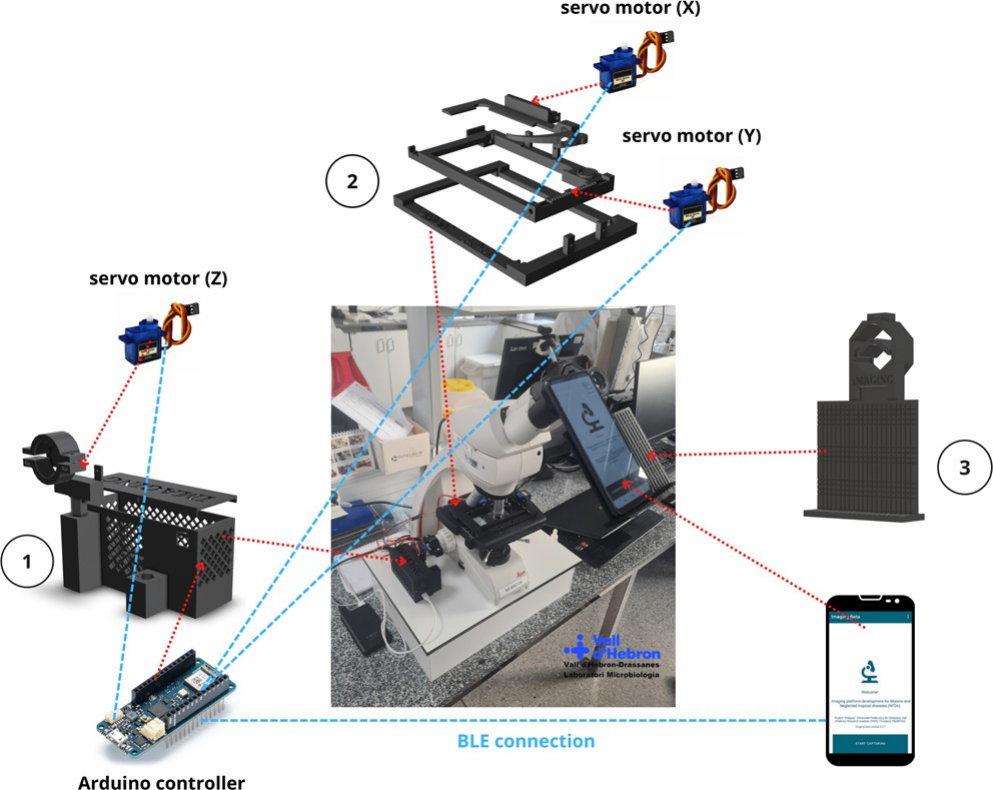
Robotized system representation. Robotized system representation on a Leica DM750 optical microscope from Microbiology Laboratory of Drassanes-Vall d’Hebron International Health Unit. Red discontinuous arrows represent space positions. Blue discontinuous lines represent connections. (1) Storage and focus 3D pieces. The Arduino controller is stored in the grid box. The servo (Z) motor is directly connected to the Arduino controller and subjected by a holder arm to change the Z position. (2) Microscope stage pieces. Three individual stage pieces were placed on the microscope stage. Two servo motors are attached to the stage 3D pieces to move the sample through the X-Y axis, and connected to the Arduino controller. (3) Mobile phone adapter pieces. The smartphone adapter is positioned on the ocular lens of the microscope. The smartphone is connected via *Bluetooth* (BLE) to the Arduino controller to guide the entire robotized procedure.

**Fig 2:**
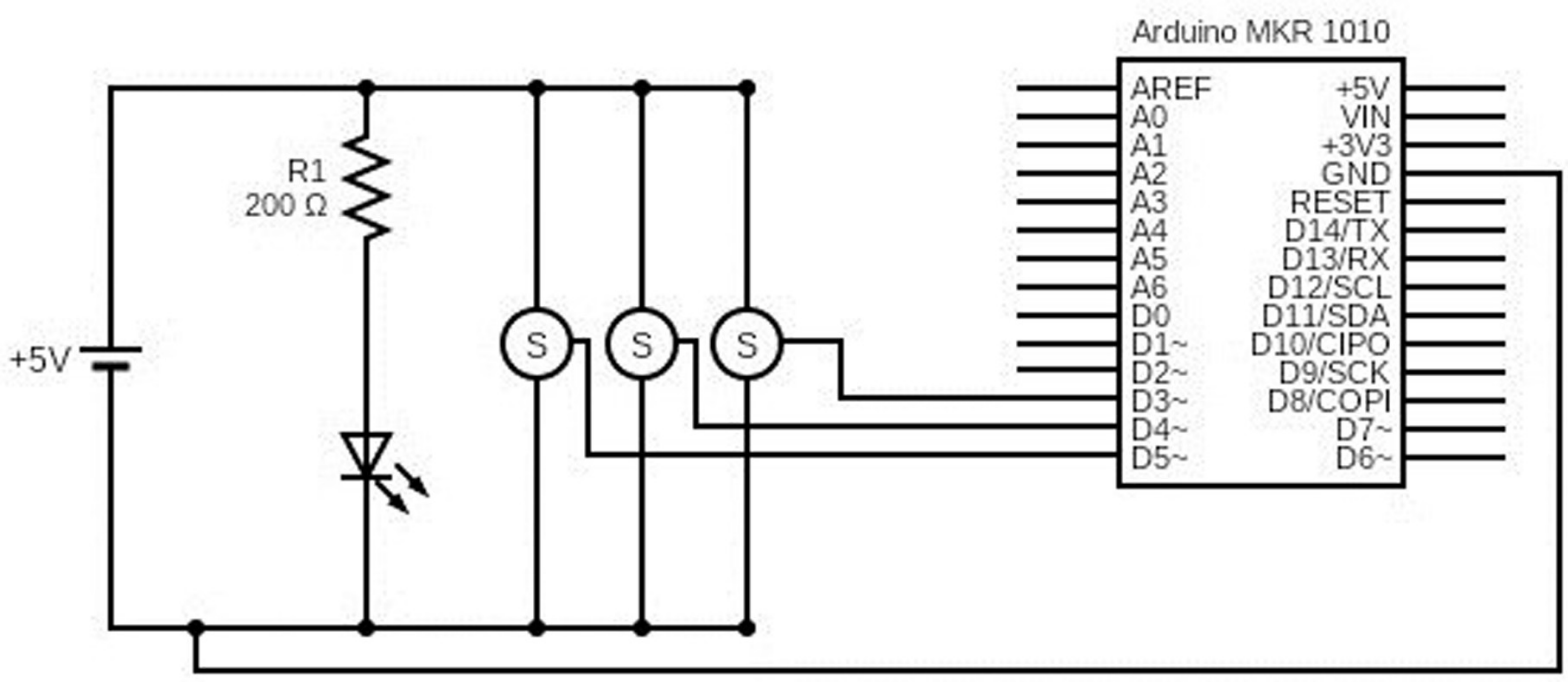
Representation of the electronic circuit and configuration. (S) Servo motor; (V) Volts; (R) Resistance; (Ω) Ohms; Ground (GND).

The system was designed to automatically replicate the movements of a conventional optical light microscope (Fig 1). It was designed as a universal prototype, which can be adapted to the majority of microscope models. Several representative microscopes were measured to define a range of measurements for building the pieces (Table 1). A 3D-prototype for the X-Y (microscope stage) and Z (focus issue) axis movements was designed. For X-Y axis, a rectangular adapter with 3 independent pieces was built. The adapter was deposited on the microscope stage, and 2 servo motors SG90 (5V, speed 0.5 seconds/120°), one for each axis X and Y, were attached to the main 3D-piece to perform the microscope movements. Two metallic bars (86 mm *length*, 3mm ⌀) were positioned between the adapter pieces to easily allow the movements of the biological sample on the microscope stage. For the Z axis focusing movements, a third SG90 servo motor was employed. All motor movements were controlled by an Arduino MKR WiFi 1010, that is controlled and connected directly via *Bluetooth Low Energy* (BLE) protocol connection to a smartphone device. The electronic diagram and configuration of the entire system is represented in Fig 2.

**Table 1:** Optical microscope measurements for the design of 3D universal adaptable pieces.

*Microscope model*	*Microscope stage dimensions* *(length x width x height)*	*Distance from the base to the micrometric wheel*	*Fine adjustment wheel*	*Ocular lens diameter*
Leica DM750	144mm x 185mm x 18mm	43mm	23mm (external diameter)	34.8 mm
Olympus CH2	152mm x 123mm x 10mm	110mm	24mm (external diameter)	31.7 mm
Nikon Eclipse E200	133mm x 220mm x 15mm	70mm	30mm (external diameter)	37.7 mm
Zeiss KF2	135mm x 125mm x 17mm	96 mm	22 mm (external diameter)	28 mm

Millimeters (mm).

### Design and manufacturing of 3D pieces

3D printing technology was employed to build the pieces for microscope automation. Tinkercad and Ultimaker Cura 5.1 slicing software were used to design and print the 3D pieces of the prototype. The designed pieces were first visualized on a Tinkercad computer viewer to determine their measurements and lengths. The designed models were exported as a *stl* file for further building and exported for slicing to Ultimaker Cura 5.1, generating a *gcode* file. Finally, pieces were printed on an Ender-3 Pro printer with PLA material. The design of the 3D parts has been specially developed for adaptation to most conventional optical microscopes. Dimensions have been empirically collected from four different representative optical microscopes to establish a range of measurements for the design of the 3D prototype parts (Table 1). The measurements of the different microscope models confer the possibility to design 3D printed pieces that could be attached in any of the analyzed microscopes. Different microscope brands (Leica, Olympus, Nikon and Zeiss) were selected in order to obtain diverse designs and measurements with representative data.

A total number of 15 individual different pieces were designed and built to create 3 main systems or subsets of pieces. Each piece has different parts with specific functions. In Table 2, all pieces’ designs are specified and classified in each of the subsets: microscope stage pieces (S4 Fig), auto-focus pieces/storage and controller parts (S5 Fig), and mobile phone adapter pieces (S6 Fig).

**Table 2:** Description of all 3D pieces employed for automated system building and assembly.

Subset pieces type	Pieces parts names	Design
**Microscope stage pieces** **(5 independent pieces)**	1. External stage holder 2. Screw hole for servo motor 3. Toothed rail for servo motor 4. Sample holder and internal stage holder 5. Medium stage holder 6. Sample clamp 7. Gears for toothed rail and servo motors	See S4 Fig
**Auto-focus pieces / Storage and controller parts (5 independent pieces)**	1. Storage box cover 2. Storage box 3. Auto-focus servo motor stick holder 4. Fine adjustment wheel clamps 5. Auto-focus system supports 6. Storage template for Arduino 7. USB hole 8. External cables hole	See S5 Fig
**Mobile phone adapter pieces (5 independent pieces)**	1. Smartphone holder 2. Adapter support 3. Ocular lens hitch 4. Wheels for regulating dimensions screws	See S6 Fig

The pieces were constructed with a 3D-printer Ender-3 Pro (Creality 3D) employing PLA material.

Three subsets of pieces were designed and constructed for microscope automation. Auto-focus pieces and storage and controller parts are encompassed in the same subset, although their individual roles are different.

#### Microscope stage pieces

**Fig 3:**
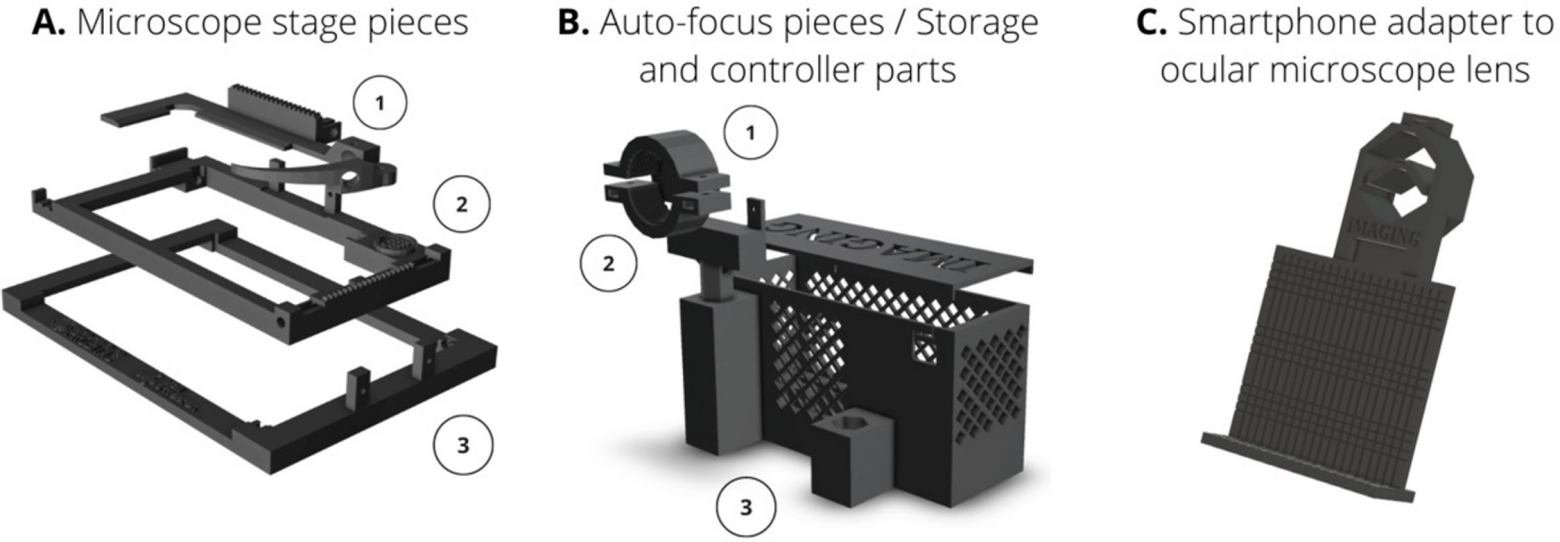
Microscope 3D pieces subsets. All pieces were designed with Ultimaker Cura 5.1 slicing and Tinkercad *softwares*. **(A)** Microscope stage pieces (1) Biological sample gripper to hold the slide. A toothed track allows for horizontal movement with the horizontal servo motor. (2) Stage holder. A toothed track allows for vertical movement with the vertical servo motor. (3) Main microscope stage piece to support the system. **(B)** Microscope auto-focus and storage pieces (1) Servo motor holder. This piece had an adjustable height to be attached on the fine adjustment wheel of the microscope. (2) Wheel holder with adjustable diameter. (3) Storage box for Arduino and board controllers. **(C)** Mobile phone adapter pieces for microscope ocular lens. An adjustable clamp allows the adapter to be attached to the eyepiece lens. The roughened PLA mount holds the smartphone device in order to capture images without moving.

Specially designed to be attached on the microscope stage. Three sub-pieces (Fig 3A) confer a single structure to hold the biological sample and move it through the X-Y axes with the assistance of the servo motors. The original holding stage clip of the microscope should be removed to place the microscope stage 3D pieces. The three sub-pieces should be correctly assembled to avoid movement issues and inaccuracies due to gaps between items. Microscope 3D stage piece should be fastened on the original microscope stage with tweezers.

#### Auto-focus pieces

A holder on the fine adjustment wheel to emulate focusing movements with the motor (Fig 3B). The pieces have an adjustable diameter to be adapted to the fine adjustment microscope wheel and moving in both directions of rotation. Two metallic screws allow the subjection of the micrometric wheel, which would be responsible of the consequent auto-focus of the sample by the smartphone camera visualization. The sample must be correctly focused for further imaging detection of objects; such as bacteria, cells, parasites or other forms.

#### Storage and controller parts

A storage cage for Arduino and motor controllers (Fig 3B). The cage was specially designed to not disturb the adaptation to the microscope. An open port for USB 2.0 to provide Arduino controller with energy power was placed on the below lateral part of the box. The storage box has a mesh with small holes to prevent the system from overheating. Inside the controller box (Fig 3B, number 3) a template with the dimensions of the controllers allows them to be stored precisely and without any possibility of displacement.

#### Mobile phone adapter

A smartphone adapter for ocular microscope lenses (Fig 3C). The adapter holds the smartphone device for the correct acquisition of microscopic images through its camera. The dimensions of the adapter were designed by the analysis of the different smartphone measurements. A PLA screw on the upper part of the holder allows to fit the adaptor to the microscope regardless of the diameter of the ocular lens.

### Optical microscope stage movements emulation by servo motors

Once the 3D pieces were designed and constructed, a set of three Micro Servo Motor 9G were used to perform the focusing and shifting microscope movements. Two micro servo motors SG90 were employed to move the biological sample through the X-Y axes, fastened by microscope stage 3D pieces. The two motors were placed horizontally and vertically respectively, on the stage adapter and attached with two metallic screws. Two metallic bars allow the movement of the PLA pieces through the X-Y axes by the micro servo motor movements. Two gearwheels are positioned on the drive wheel of the servo motors, and by means of toothed rails allow movement in both X-Y directions. Both servo motors were controlled by the Arduino MKR WiFi 1010, connected by BLE connection to the smartphone device. One servo motor was employed to move the fine adjustment wheel of the microscope. The motor was subjected with a 3D PLA piece with a rounded hole fitting the form of the engine, and a hexagonal base stick (Fig 3B, number 1*)*. A circular screw with an adaptable dimension was attached to the servo motor. The screw (Fig 3B, number 2) holds the fine adjustment and moves it in both rotation directions. The auto-focus device was positioned on the storage box, conferring the system a more compact structure.

### Auto-focus algorithm

An image auto-focusing method was designed to correctly acquire readable and focused images for further image analysis. The microscope must be in a focused position to capture an optimal image with the smartphone device. In our mechanism, we have employed the variance of Laplacian as a reference method for image auto-focusing. The variance of Laplacian allows the calculation of a value for each image, which indicates the level of focusing of the acquired picture. Focused images have higher values of variance of Laplacian than blurred images. Therefore, the analysis of variance of Laplacian values would determine which is the best focused image for each Field of View (FoV).

The calculation of the variance of Laplacian is specified in *Eq 1*; where Δ is the Laplacian operator (*Eq 2*) applied to an image *I*(*m*,*n*) by convolving a 3 x 3 Laplacian kernel; and $\bar{\Delta I}$ is the average of the Laplacian (*Eq 3*) [11].


$$VAR_{-}LAP_{m,n}=\sum_{i}^{m}\sum_{j}^{n}{\left[|\Delta I(i,j)|-\bar{\Delta I}\right]}^{2}$$

*Equation 1*: The calculation of the variance of Laplacian (VAR_LAP).


$$\Delta (I)=I(m,n)*\left[\begin{array}{ccc} 0 & 1 & 0\\ 1 & -4 & 1\\ 0 & 1 & 0 \end{array}\right]$$

*Equation 2*: The calculation of the Laplacian operator by convolving the image *I*(*m*,*n*) to the 3 x 3 kernel.


$$\bar{\Delta I}=\frac{1}{m\times n}\sum_{i}^{m}\sum_{j}^{n}|\Delta I|$$

*Equation 3*: The calculation of the average of the Laplacian. The image (I) has “*m*” (width pixels) and “*n*” (height pixels) of dimensions.

**Fig 4:**
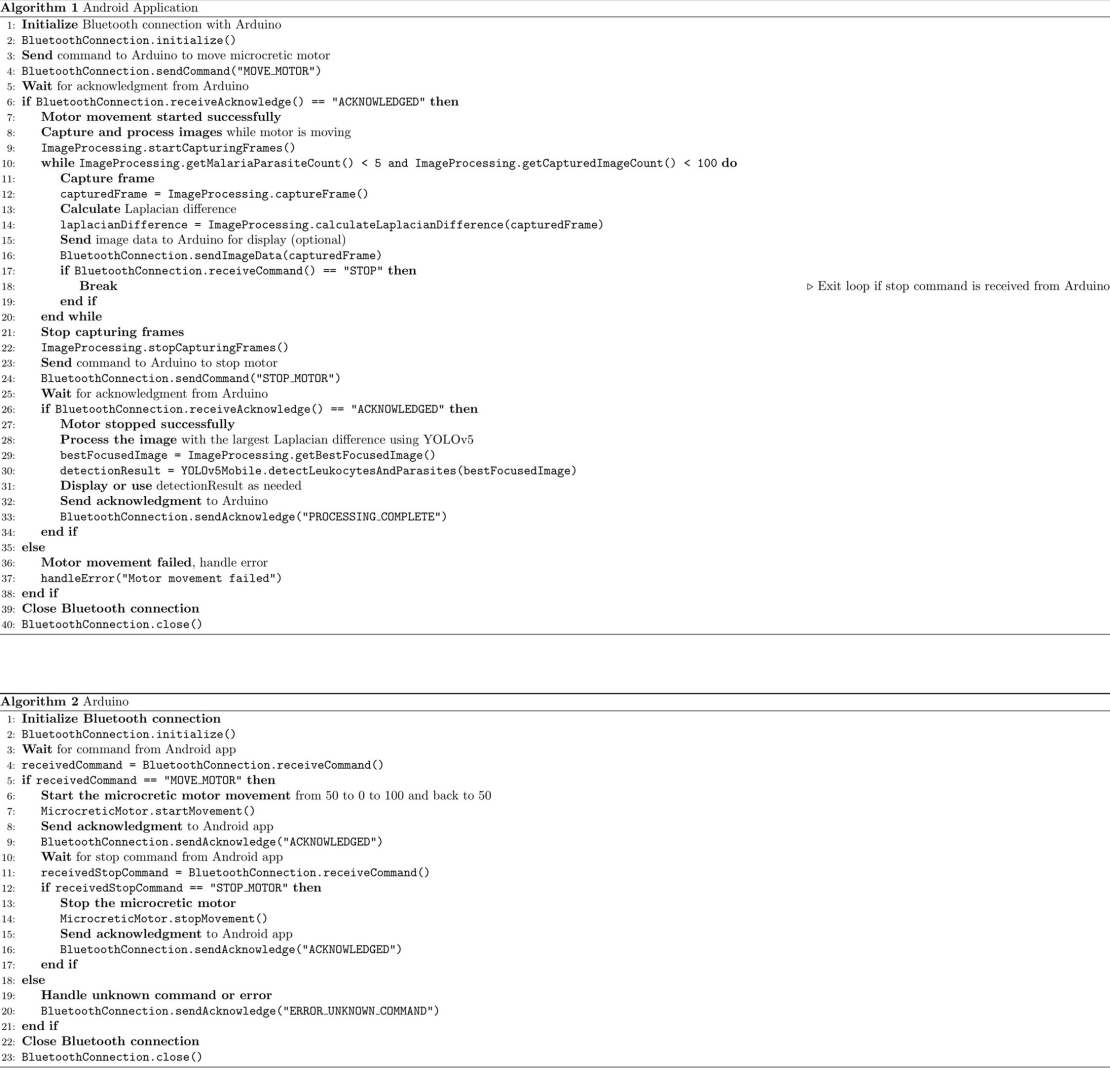
Pseudo-code. Pseudo-code of the android application settings (algorithm 1) and arduino controller (algorithm 2).

The variance calculation is performed in each FoV determined by X-Y movements of the robotized microscope. In a single FoV, the smartphone camera observes different focused images (Z axis) by the continuous movement of the Z-servo motor on the fine adjustment wheel. The smartphone device by *BLE* connection with the controllers guides the Z-servo motor to move the fine adjustment wheel in both directions of rotation [50 position units of movement (u.m.) in each direction; 1 u.m. = 1°] in order to focus the biological sample. Pseudo-code details are represented in Fig 4. During the auto-focusing process a Laplacian variance value is computed to each of the images/frames of a real-time video. In order to avoid unfocused positions, the system visualizes the centroid of the original image by creating a new cropped image with 50% width and 33% height image for Laplacian analysis. In addition, a 40 milliseconds delay was added to correctly acquire the images. This procedure allows the observation of only the center of the image, without the black borders produced by the ocular lens attachment and the blurred edges. Once the image scanning in the two directions of rotation has been completed, the mechanism is able to obtain the image with the highest Variance of Laplacian value for further image analysis.

### Ethics statement

This study was conducted in accordance with the Declaration of Helsinki and approved by the Clinical Research Ethics Committee (CEIm) of the Vall d’Hebron University Hospital/Vall d’Hebron Research Institute with reference number PR(AG)40/2023.

## Results

### Universal system adaptation

The developed system is specially designed to be adaptable to most conventional optical microscopes. The size measurements collected from optical microscopes of different brands and models allows the design of a universal adaptable system (Table 1). Each of the subgroups of 3D parts has been designed for a range of measurements within the dimensions of the microscopes analyzed. The microscope stage mounted pieces have 141 x 100 x 7.5 mm dimensions. Final measures of length and width can be adjusted to any of the analyzed microscopes. The height dimension of 7.5mm was designed to be as small as possible in order to not interfere with the stirring of objective lenses of the microscope. The sample slide is in direct contact with the stage of the original microscope, in order to emulate a conventional microscopic visualization and do not modify the distances between the objective lenses and the biological sample. Auto-focus pieces have a range of diameters of the coupling hitch with a micrometric wheel of 20–30 mm. In order to attach the auto-focus pieces from the base to the fine adjustment wheel, two vertical holder pieces were built with a range of measures of 38–48 mm length. Smartphone adapter pieces have a range of measures of 25–50 mm length, regulated by a metallic screw, on the ocular lens attachment. The specific dimensions of the prototype are summarized in S1–S3 Figs, and 3D pieces’ models are open-source and publicly available in *supplementary material* section as *stl* files.

### Robotized and automated slide scanning

**Fig 5:**
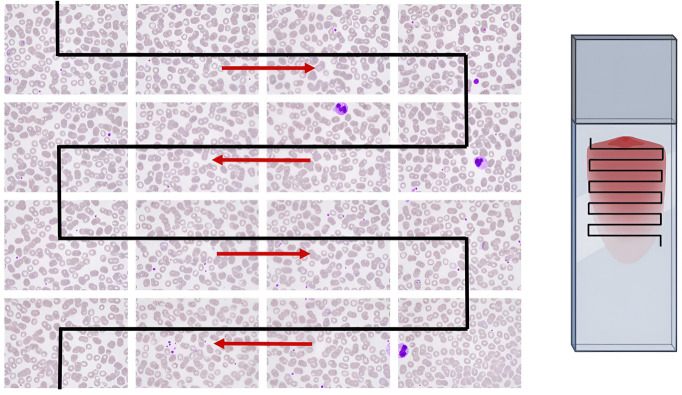
Sample scanning X-Y movement representation. Automated snake-like movement of the system to capture and scan the whole blood smear samples. Automated scanning strategies using imaging techniques for object detection through X-Y axes. Snake-like movement for image acquisition of a rectangular shape sample. Images from Microbiology Laboratory of Drassanes-Vall d’Hebron International Health Unit.

A movement pattern has been developed which can be adapted to the needs of observation according to the type of biological sample. Horizontal and vertical movements through the X-Y axis of the sample slide are crucial to scan the maximum area in order to detect pathogens of interest or morphologies. The biological sample is placed on the glass slide and usually has a rectangular, circular or square shape, although sometimes the sample has an irregular form. It is crucial to only observe the area of interest, avoiding non-stained or empty spaces in the slide. The most efficient scanning procedure to observe the maximum surface of a rectangular or square shaped sample is a snake like movement from left to right and from the top to the bottom (Fig 5). To scan a single FoV with 100x objective magnification, servo motors perform the snake-like movement every 5 units of movement (u.m.) (Operating Speed 0.12 sec/60°). The system stops in each FoV in order to acquire a new image with unseen information. The system allows the selection of the sample shape and the magnification of the observation in order to move the sample and capture images correctly.

### Auto-focus scanning

The Arduino is connected via *BLE* to the smartphone and applies the variance of Laplacian algorithm to auto-focus the sample. It is necessary to help the system with the first focus in order to reduce diagnosis time by the observation of a blurred image on smartphone screen. Once the first focus was performed, the system initiated the auto-focus of the sample in each microscope FoV, by the observation of a real-time video. Biological samples are not perfectly plane and have a slight relief that might affect the focus of each FoV. Therefore, the first auto-focus is wider (50 u.m. of the servo motor) in comparison with the following auto-focus movements of the subsequent FoV (30 u.m. of the servo motor). This procedure allows the process to be faster, avoiding delays due to autofocusing of individual microscopic fields.

**Fig 6:**
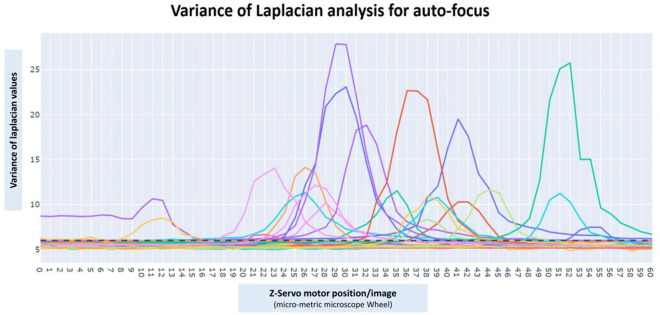
Variance of Laplacian values for auto-focus system analysis. A total number of 30 microscopic fields (images) of 6 different Giemsa stained thick blood smear samples (5 FoV/sample) for malaria diagnosis were analyzed. Each FoV has a range of Variance of Laplacian values depending on the position/image [60 total positions (30° on each side)] of the fine adjustment microscope wheel. Focused images were represented as the higher Variance of Laplacian values (peaks correspond to in-focus positions/images).

In order to evaluate the performance of the proposed auto-focus algorithm, an analysis experiment was designed. Auto-focus evaluation was described by the observation of different FoV of Giemsa stained thick blood smear samples (n = 6) at 1000x total magnification for malaria diagnosis, with a Zeiss KF2 microscope. A total number of 5 FoV images of each sample were acquired (30 FoVs in total). The observation was emulated with the digital camera of a Samsung Galaxy A13 smartphone device. Variance of Laplacian analysis was performed in each FoV while the servo motor moves the fine adjustment wheel of the microscope in order to find a focused image/frame of the real-time video. A total number of 60 micro-metric positions (30° on each side) were postulated in order to scan different focused images of the same FoV. The system determines the image/frame linked with the higher Variance of Laplacian value (most focused image), in order to capture it for further CNN analysis. Time of focus and Variance of Laplacian value were analyzed. Results were presented in Fig 6 and Table 3. An average time of 27.00 ± 2.58 seconds was required to auto-focus a single FoV with the described technology. Variance of Laplacian values of ≤ 6 are considered unfocused or empty FoVs. Images with non-biological material or stain (transparent) were not optimal for autofocusing and analysis. Results demonstrate a mean average maximum Laplacian value of 11.83, representing focused images for further CNN image analysis.

**Table 3:** Autofocus analysis of thick blood smear Giemsa stained.

Sample Number	Field Number	Time of Focus (milliseconds)	Laplacian Variance (maximum)	Position/Image (0–60)
P1	1	23.131	7,47	54
P1	2	25.116	10,28	41
P1	3	26.123	11,52	35
P1	4	29.878	10,63	11
P1	5	27.451	14,13	26
P25	1	27.584	11,33	26
P25	2	30.082	5,5	10
P25	3	25.657	8,33	38
P25	4	27.213	10,2	28
P25	5	29.767	8,5	12
P26	1	25.177	19,5	41
P26	2	28.167	6,1	22
P26	3	24.161	6,01	47
P26	4	26.584	18,83	32
P26	5	31.504	6,01	1
P19	1	23.428	11,24	51
P19	2	31.620	6,01	1
P19	3	24.454	11,61	44
P19	4	27.967	14,1	23
P19	5	30.837	6,39	5
N3	1	27.046	23,1	30
N3	2	26.003	22,66	36
N3	3	23.273	25,75	52
N3	4	26.959	27,85	29
N3	5	31.222	5,73	3
P40	1	25.405	10,8	39
P40	2	28.174	6,66	22
P40	3	22.830	5,41	55
P40	4	27.445	12,14	27
P40	5	25.721	10,96	38
*MEAN*		*26*.*999*	*11*,*83*	

Giemsa stained samples (n = 6). Time of Focus, Laplacian Variance and Position/Image values were represented.

System features were represented in Table 4. Several state-of-the-art published systems were selected in order to be compared with our purpose in terms of: pieces materials, image acquisition, autofocus, cost, camera settings, point of care design and power requirements [13, 17–20].

**Table 4:** Summary table comparison of the state-of-the-art automated microscope designs.

Design	Pieces materials	Automated image acquisition	Autofocus (focusing time)	Approximate cost	Camera	Point of care design	Power requirements / Internet connection	Additional requirements
Delahunt et al 2015 [17]	Aluminium	Yes	Yes. Brenner gradient algorithm (Z-stack)	1500$-4000$	MIGHTEX camera	Yes	15.8W / Non-specified	
Veerendra et al 2015 [20]	Non-specified	Yes	Non-required	Low-cost (non-specified)	Samsung Galaxy camera (EK-GC100)	Yes	Non-specified / Non-needed	Microfluidic-based system
García-Villena et al 2021 [18]	Polylactic acid (PLA) (3D printing)	Yes	Manual focus	Low-cost (non-specified)	Samsung S9 camera (minimum pixel resolution of 1440 × 1080)	Yes	Stepper drivers (12 V), LED light (3W) / Non-required (desirable)	Smartphone device
Oyibo et al 2022 [13]	Aluminium profiles (3D printing)	Yes	Yes. Greyscale 2D Laplacian filter.	Low-cost (non-specified)	Raspberry Pi High-Quality Camera Module V2.1, equipped with a Sony IMX477R	Yes	60W / Non-specified	
Gordon et al 2022 [19]	Aluminium and acrylonitrile butadine styrene (ABS) (3D printing)	Yes	Yes (algorithm non-specified)	1300$	1.6 MP monochromatic Blackfly S camera with a Sony IMX273 sensor	Yes	Non-specified / Non-required	Microfluidic-based system. Fluorescent stain.
de Oliveira et al 2024	Polylactic acid (PLA) (3D printing)	Yes	Yes. Laplacian variance (27.00 ± 2.58 seconds)	220–300 $ + (300$ smartphone) = 520$-600$	Samsung Galaxy S20 camera (minimum 5MP)	Yes	10W / Non-needed	Conventional optical microscope + smartphone device

## Discussion

According to our knowledge, it is the first fully low-cost adaptable automated system for infectious diseases diagnosis. These characteristics are one of the major strengths of the project, and facilitates the implementation of the system in any clinical laboratory environment. Out-of-measures microscopes could also be adaptable for our system, although new pieces should be designed for its correct implementation. In addition, it is also compatible with microscope systems containing an integrated camera. However, many of the published automated systems are based on a single design, and therefore make them difficult to implement in any laboratory due to their low adaptability. However, the acquisition of complete systems such as EasyScan Go could complement traditional laboratory tools with promising results [21]. Our system is low-cost ($220-$300) due to its manufacturing materials and servo motors; its power requirements (10W) are accessible; does not need internet connection; has an efficient auto-focus technology; and is specially designed for resource-poor settings implementation. However, some of the limitations of the system are the conventional microscope requirement for its operation, the non-automated change of magnification, and the need of an external camera (smartphone). Other designs described in Table 4, such as Oyibo et al 2022 do not need a conventional optical microscope for its functioning, although its power requirements are higher and therefore it is not an adaptable design [13]. García-Villena et al 2021 purposes a low-cost portable prototype specially design for its implementation in resource-poor settings, although it requires manual focusing [18]. Moreover, Gordon et al 2022 and Veerendra et al 2015 designed similar alternatives based on microfluidic technology for diagnosis and object detection.

Diagnosis procedures by microscopic visualization of biological forms are different depending on the type of observed sample and disease. For example, in the case of malaria disease, the observation of *Plasmodium* parasites in thick and thin blood smear samples with a total magnification of 1000x (100x immersion oil + 10x ocular lens) is crucial to perform a correct diagnosis [22]. Moreover, it is not necessary to observe the whole sample to perform a diagnosis, and consider a positive/negative result for *Plasmodium* infection. If a single parasite is observed it is considered as a positive diagnosis, although if the microscopist observes enough microscope fields (at least 100) without any parasite observation, the sample is considered as a negative result [23]. However, for the diagnosis of other parasitic diseases such as *Schistosoma haematobium* in urine sediment samples, the whole sample should be observed in order to detect the eggs at 100x and 400x total magnification (10x and 40x objective lens + 10x ocular lens) [13]. These differences in the observation methodologies and procedures are quite common in microscopy, and should be considered for the development of automated diagnostic methods. With our system, the snake-like path of the X-Y servo motors allows its movements to be adaptable to most types of microscopic clinical specimens.

Autofocusing the sample for the correct acquisition of images is crucial to perform a correct image analysis and automated diagnosis. Variance of Laplacian technique is widely used as a reference method to evaluate the autofocus of digital images in other studies [24–26]. However, focus time is an issue when quickness and effectiveness for microscopy diagnosis is a requirement. As an alternative to our work, Bueno-Ibarra et al. proposed a fast auto-focus algorithm for automated microscopes by Fourier Transform and Pearson correlation [27]. The auto-focus algorithm requires time of analysis and the correction of the focus in each FoV slows down the acquire process. The morphology of biological samples should be correctly interpreted, assuming three dimensions of observation. The height or Z dimension of a sample on a microscope slide is variable and often irregular, since the disposition of the cells, bacteria or staining reagents create a relief on the slide that would consequently affect the autofocus of the sample in each FoV. In addition, preprocessing techniques such as noise reduction, background correction, contrast enhancement and image cropping could help to eliminate undesirable artifacts or effects that would affect image focusing [11]. However, these methodologies would increase even more the time of acquisition and analysis.

It is crucial to determine a balance between time and image quality in order not to obtain diagnoses that are too slow, or incorrectly captured images that hinder the object detection and classification performed by the CNN systems.

### Diagnostic AI-based applications and perspectives

The developed automated system can be combined with artificial intelligence-based convolutional neural network models to perform fully autonomous diagnostics. Artificial Intelligence (AI) is one of the most outbreaking developing technologies during the current century. Improvements in deep learning techniques will allow the development of better and new AI applications in several research topics. European Parliament published recent studies related with AI in diplomacy, environmental impact green policies, open-source approaches, capital flows or data availability [28]. Artificial intelligence, in nowadays years, has played a crucial role in diagnostic support and biomedical research, and should become even more important in the near future. As some examples, COVID-19 computer-aided diagnosis by classification of CT images with deep learning models [29]; AI algorithms for the prognosis, diagnosis and treatment selection for precision oncology improvements [30]; machine learning and statistical techniques for differentiating tropical infectious diseases such as malaria, dengue and leishmaniasis [31]; or an automated microscopy for the diagnosis of *Schistosoma haematobium* eggs in resource-poor settings by AI techniques [13], are some of the main applications of this promising technology.

As discussed in the introduction, the development of novel diagnostic techniques to solve microscopy issues and improve resource-poor settings environments by its implementation will be a major challenge for the following years. Therefore, the low-cost automation of a standard microscope presented in this study in combination with image AI-based diagnosis tools would be an excellent alternative in these contexts. Evaluation of state-of-the-art CNN algorithms for malaria parasite detection in thin blood smear samples demonstrate a 97% of accuracy to distinguish between an infected and an uninfected erythrocyte [32]. Literature shows a wide variety of systems based on AI able to detect malaria parasites in digital images, in order to support and complement traditional microscopy [7, 22, 32–37]. Our research group trained multiple smartphone-based computational state-of-the-art deep learning models for malaria parasite detection in thick blood smear digital images and tested the robotized *iMAGING* prototype with promising results [38]. This work was part of the same project presented in the manuscript, in which AI algorithms were a complement to the low-cost automation system and the smartphone device application. A dataset of 2571 annotated digital images of thick blood samples were employed. Comparative analysis yielded a performance for YOLOv5x on a test set of 92.10% precision, 93.50% recall, 92.79% F-score, and 94.40% mAP0.5 for leukocyte, ring stage and mature *Plasmodium* trophozoites overall detection. F-score values of each category were 99.0% for leukocytes, 88.6% for early trophozoites and 87.3% for mature trophozoites detection [38].

**Fig 7:**
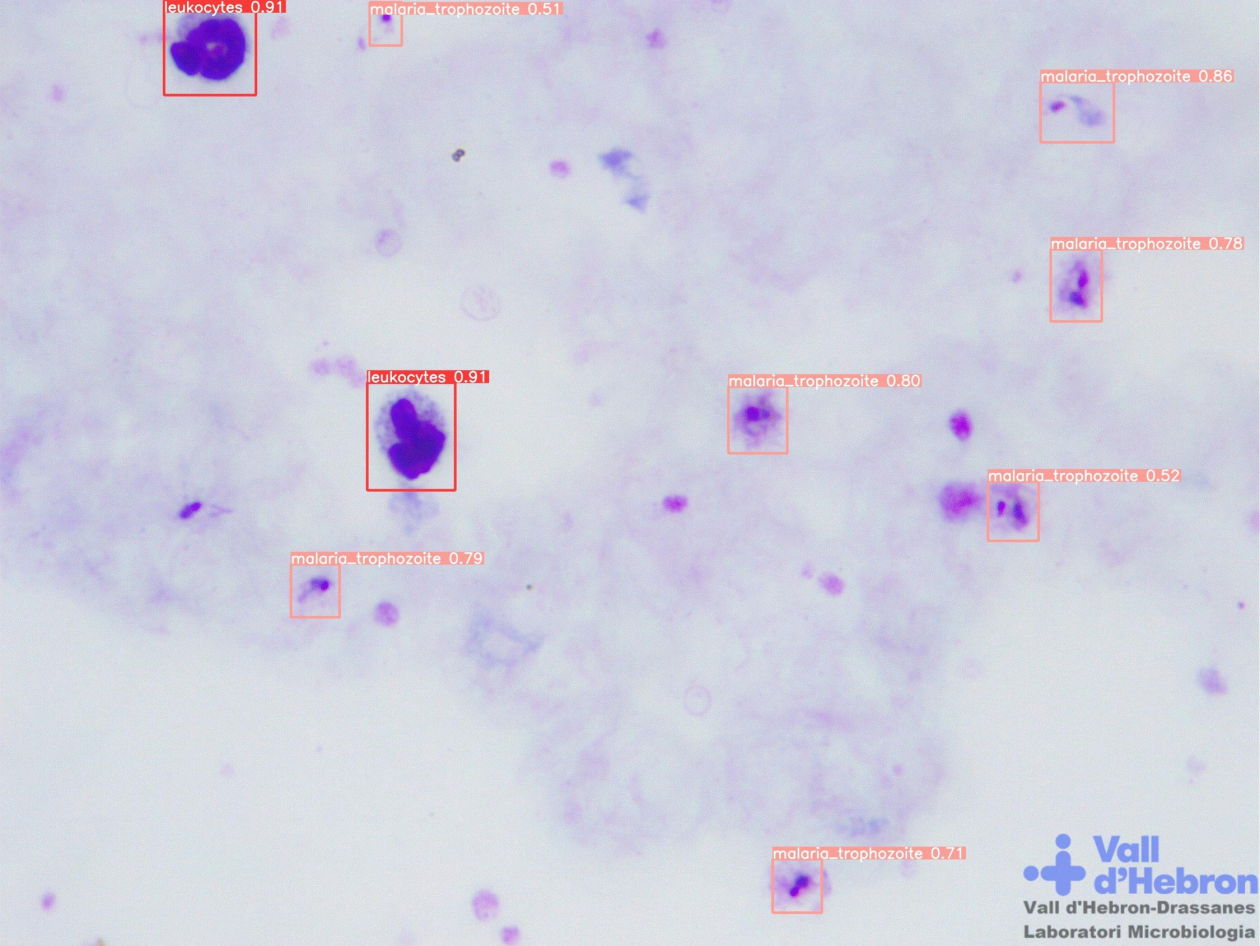
Microscopic images from Microbiology Laboratory of Drassanes-Vall d’Hebron International Health Unit. Giemsa stained thick blood smear sample with detection of leukocytes and malaria trophozites by YOLOv5x neural network performance. 1000x magnification.

**Fig 8:**
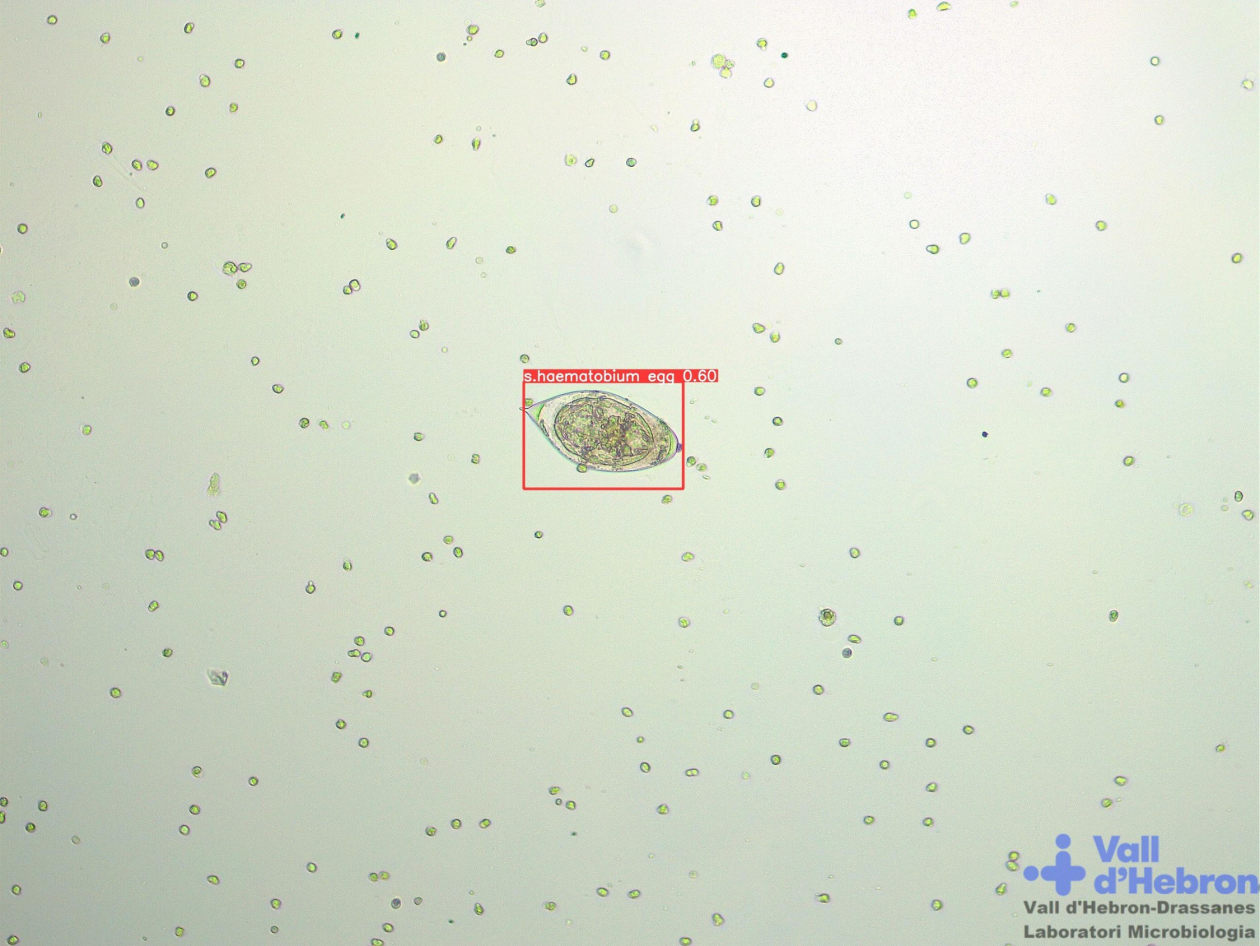
Microscopic images from Microbiology Laboratory of Drassanes-Vall d’Hebron International Health Unit. Urine sediment sample with detection of *Schistosoma haematobium* eggs by YOLOv8x neural network performance. 100x magnification.

Moreover, schistosomiasis diagnosis is mainly based on the visualization of parasite eggs in stool (Kato-Katz technique) or urine sediment samples by microscopic examination [39]. As an alternative, Schistoscope system is an optical diagnostic device for the automated detection of *Schistosoma haematobium* eggs through the X-Y-Z-axis movements for sample scanning. A robust image dataset containing over 5000 FoV images of filtered spiked and clinical urine samples was employed for the generation of AI models for image analysis detection [13]. Our research group has assessed if our developed system is able to detect *Schistosoma haematobium* eggs in automatically acquired images with YOLOv8x neural network showing 95.3% precision, 89.9% recall, 92.5% F-score and 96.8% mAP0.5. Example images of thick blood smear (Fig 7) and urine sediment samples (Fig 8) analysis are represented.

As another example, Soil-transmitted helminthiasis (STH) is an NTD caused by intestinal parasitic worms transmitted through contaminated soil. Eggs of *Ascaris lumbricoides*, *Trichuris trichuria*, *Ancylostoma duodenale* and *Necator americanus* are passed in the feces of infected people, and could be observed by optical microscopy for diagnosis. Ward et al. 2022 prototyped an AI device to detect helminth eggs and *Schistosoma mansoni* eggs in stool. The system achieved an average precision of 94.9 ± 0.8% and recall of 96.1 ± 2.1% for helminth egg species detection [40]. In addition, american trypanosomiasis or Chagas disease is a NTD produced by the protozoan parasite *Trypanosoma cruzi*. During the acute phase of infection, Chagas disease diagnosis is performed by the direct microscopic observation of *Trypanosoma cruzi* parasite in blood smears. Morais et al. 2022 developed an automated system for the detection of *Trypanosoma cruzi* parasites in blood smears using machine learning algorithms applied to smartphone images. The final system demonstrates a final precision of 87.6%, and sensitivity of 90.5%, illustrating promising results for diagnosis [41]. Finally, Leishmaniasis is a disease caused by more than 20 species of *Leishmania* protozoan parasite. Microscopy diagnosis to detect amastigotes in giemsa-stained lesions of scrapping, biopsies, or impression smears are commonly employed as a reference diagnostic technique [42]. Zare et al. 2022 developed a machine learning-based system for detecting leishmaniasis parasites in microscopic images. The Viola-Jones algorithm was employed for parasite detection, with 50% precision and 65% recall for infected macrophages detection; and 71% precision and 52% recall for amastigotes outside macrophages detection [43].

## Conclusions

A robotized system for automated diagnosis with a conventional microscope and a standard smartphone was fully developed for its low-cost implementation in resource-poor setting laboratories. Microscope robotization is a crucial step for automated diagnosis based on AI technology. Without the robotized movements of the microscope the entire process would not be completely autonomous. In addition, the design and implementation of a universally adaptable system should be affordable for any laboratory, regardless of their resources. Low-cost materials and a simple process make our system a valuable technology. Moreover, auto-focus movements are not commonly implemented in low-cost and conventional microscopes for AI detections. Finally, Z-movements confer the system a fully automated analysis without the mechanical support of a human. The combination of hardware, low-cost materials and accessible technology, plus the adaptation of the 3D-pieces to a conventional optical microscope and the use of smartphone devices, gives the system the innovative nature required for its implementation in the field. Moreover, the freely available 3D-pieces designs provide the scientific community with open-source prototypes for its improvement and usability.

The digitalization of diagnosis would be the next step for the autonomous diagnosis worldwide. The standardization of automated diagnosis procedures should be addressed to provide reliable results and increase its efficiency [44]. Furthermore, the generation of data from microscopic images would help to generate databases for further AI algorithms training. The coalescence of autonomous movements and image analysis provides a milestone for the implementation of available automatic diagnosis with conventional optical microscopes [45].

In conclusion, we are ever closer to develop a fully automated system to perform autonomous microbiological diagnosis emulating traditional microscopy techniques. After successfully implementing the low-cost automation of a conventional optical microscope, the irruption of AI technology for the diagnosis during the near years allows us to postulate that convolutional neural networks for image analysis will be improved, regulated and optimized to be considered as a reference diagnosis technique for malaria and NTDs detection.

## Supporting information

S1 Table List of materials and devices employed for the development, design and manufacturing of the robotized conventional optical microscope system. (DOCX)

S1 Fig Microscope stage 3D pieces dimensions representation. (TIFF)

S2 Fig Mobile phone adapter 3D pieces dimensions representation. (TIFF)

S3 Fig Auto-focus 3d pieces / Storage and controller parts dimensions representation. (TIFF)

S4 Fig Microscope stage pieces (5 independent pieces). (1) External stage holder; (2) Screw hole for servo motor; (3) Toothed rail for servo motor; (4) Sample holder and internal stage holder; (5) Medium stage holder; (6) Sample clamp; (7) Gears for toothed rail and servo motors.
(TIFF)

S5 Fig Auto-focus pieces / Storage and controller parts (5 independent pieces). (1) Storage box cover; (2) Storage box; (3) Auto-focus servo motor stick holder; (4) Fine adjustment wheel clamps; (5) Auto-focus system supports; (6) Storage template for Arduino; (7) USB hole; (8) External cables hole.
(TIFF)

S6 Fig Mobile phone adapter pieces (5 independent pieces). (1) Smartphone holder; (2) Adapter support; (3) Ocular lens hitch; (4) Wheels for regulating dimension of the screws.
(TIFF)

S1 Data (ZIP)
