# Supplementary material for: Development of a low-cost robotized 3D-prototype for automated optical microscopy diagnosis: An open-source system
Source: PLoS One. 2024 Jun 21;19(6):e0304085. doi: 10.1371/journal.pone.0304085 (PMC11192333; doi:10.1371/journal.pone.0304085)
Supplement: S1 Table — (DOCX) [file pone.0304085.s001.docx]

**S1 Table. List of materials and devices employed for the development, design and manufacturing of the robotized conventional optical microscope system.**

| **Material / Device** | **Specifications** |
| --- | --- |
| Conventional optical microscope | Binocular, with quadruple revolving objectives of x4, x10, x40 and x100 (immersion oil) magnification; and an ocular lens of x10 magnification. Fine and coarse adjustment wheels to focus the sample through the Z-axis, a microscope stage to deposit the sample with motion along X-Y axes, and light illumination. |
| 3D-printer Ender-3 Pro (Creality 3D) | Filament diameter: 1,75 mm. Print volume: 220mm x 220mm x 250mm. 240V and 270W. Polylactic Acid (PLA): an easy-to-use, low-cost, biodegradable, and recyclable material.  **Not mandatory** |
| Micro Servo Motor 9G (**x3**) | Dimensions: 22mm x 11.5mm x 22.5mm; Net Weight: 9 grams; Operating speed: 0.12second/ 60degree ( 4.8V no load); Stall Torque (4.8V): 17.5oz /in (1kg/cm); Temperature range: -30 to +60; Dead band width: 7usec; Operating voltage: 3.0V~7.2V; Coreless motor; All nylon gear; Dual ball bearing; Connector wire length 150mm |
| Arduino MKR WiFi 1010 | Based on SAMD21 Cortex®-M0+ 32bit low power ARM® MCU microcontroller. Board power supply (USB/VIN) 5V; circuit operating voltage 3.3V; DC Current per I/O Pin 7mA; SRAM 32 KB; CPU Flash Memory 256 KB (internal); Full-Speed USB Device and embedded Host; Length 61.5 mm; Width 25mm; Weight 32 grams. |
| Samsung Galaxy S20 | 2.73 GHz Octa-Core CPU; 158.5 mm / 154.1 mm screen; 3200 x 1440 resolution Quad HD+; multiple resolution rear camera (12MP + 64 MP + 12MP), (F1.8, F2.0, F2.2), autofocus, OIS, Hybrid Optic Zoom 3x to 30x, 10MP; RAM 8GB; internal memory 128 GB; Bluetooth v5.0. |
| Xiaomi Redmi 10C | 4 x 2.4 GHz Kryo 265 Gold Octa-Core CPU; 169.6 x 76.6 x 8.3 mm; 720 x 1650 pixels resolution; rear camera 50MP F/1.8 26mm (wide), 2MP F/2.4 (depth), HDR; RAM 4GB; internal memory 64 GB; Bluetooth v5.0. |
| Samsung Galaxy A13 | 4 x 2.0 GHz Cortex-A55 Octa-Core CPU; 165.1 x 76.4 x 8.8 mm; 1080 x 2408 pixels resolution; rear camera 50 MP F/1.8 (wide), 5MP F/2.2 (ultrawide), 2MP F/2.4 (macro), 2MP F/2.4 (depth), HDR; RAM 6GB; internal memory 128 GB; Bluetooth v5.0. |
