# Supplementary figures and images for: Development of a low-cost robotized 3D-prototype for automated optical microscopy diagnosis: An open-source system
Source: PLoS One. 2024 Jun 21;19(6):e0304085. doi: 10.1371/journal.pone.0304085 (PMC11192333; doi:10.1371/journal.pone.0304085)

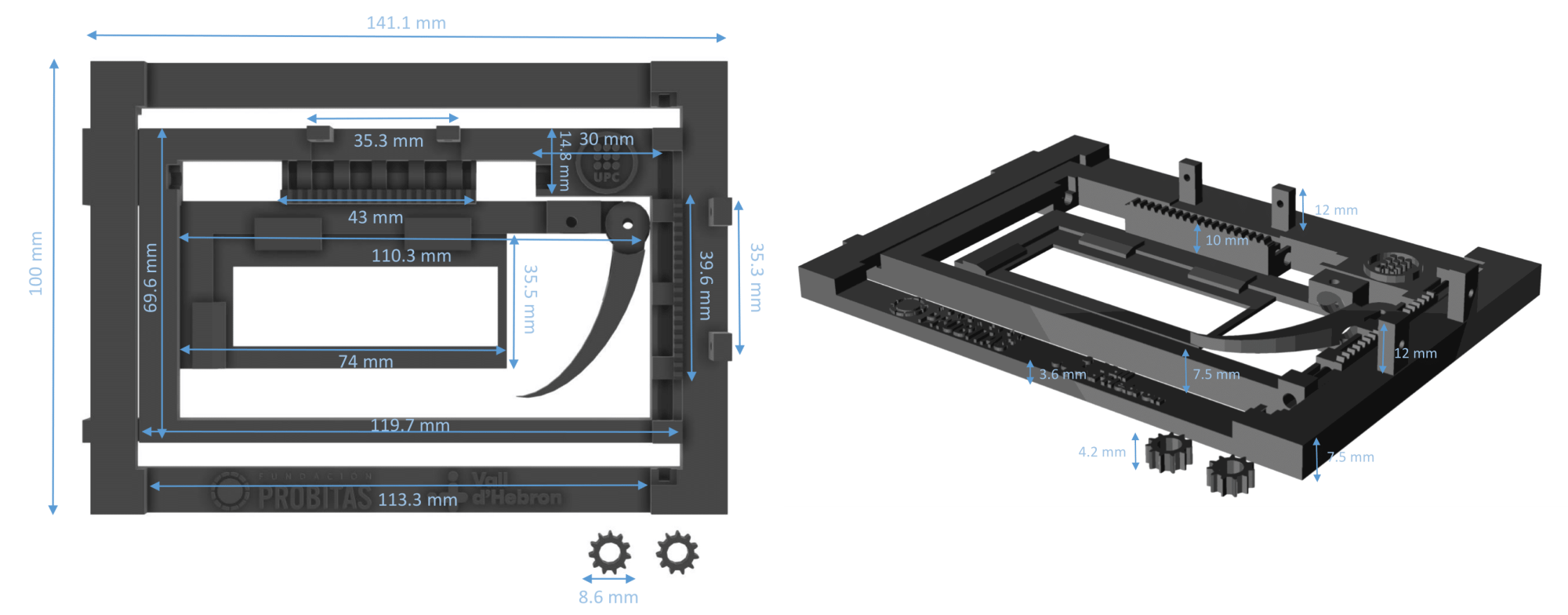

Supplement: S1 Fig — (TIFF) [file pone.0304085.s002.tiff]

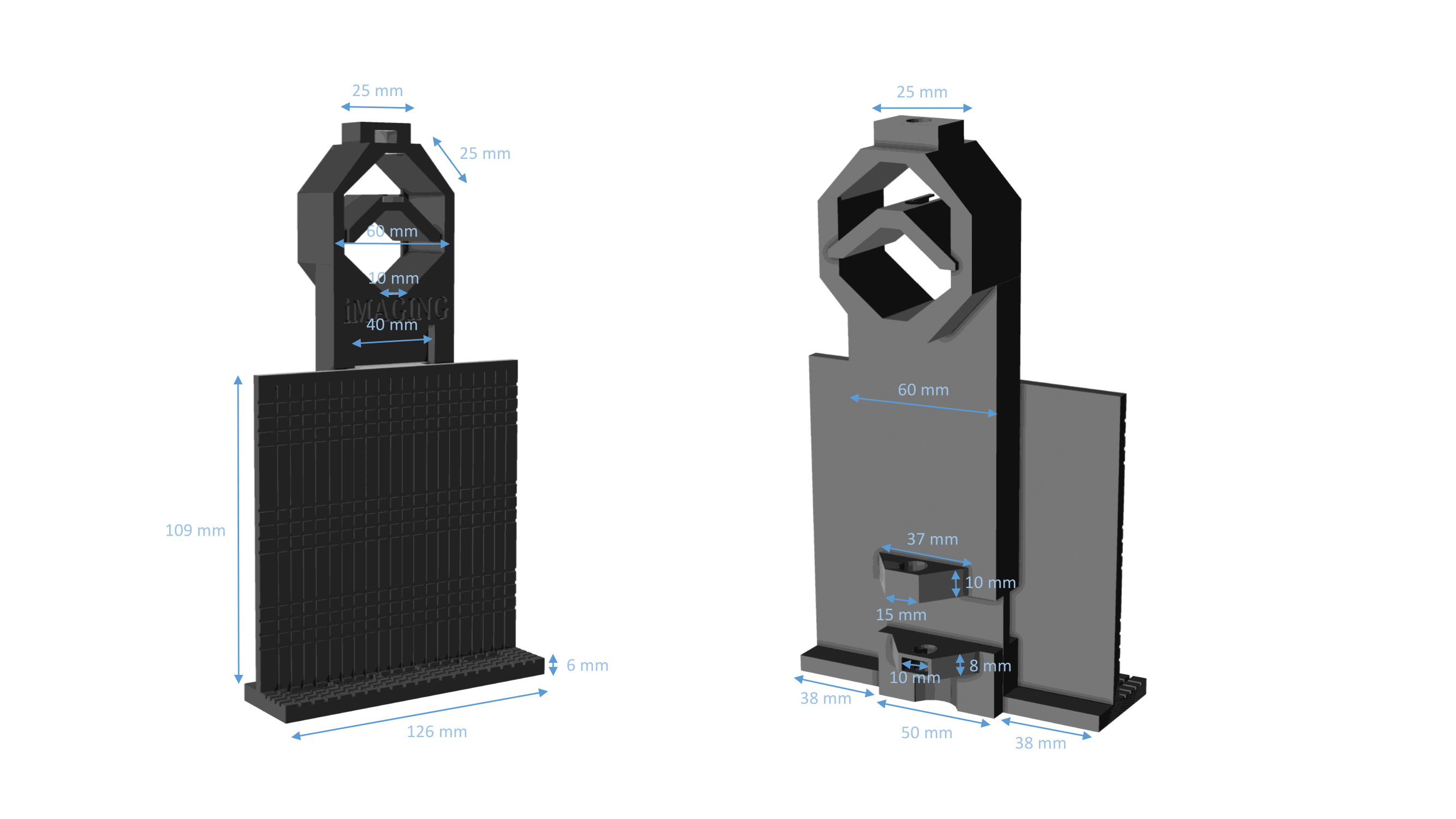

Supplement: S2 Fig — (TIFF) [file pone.0304085.s003.tiff]

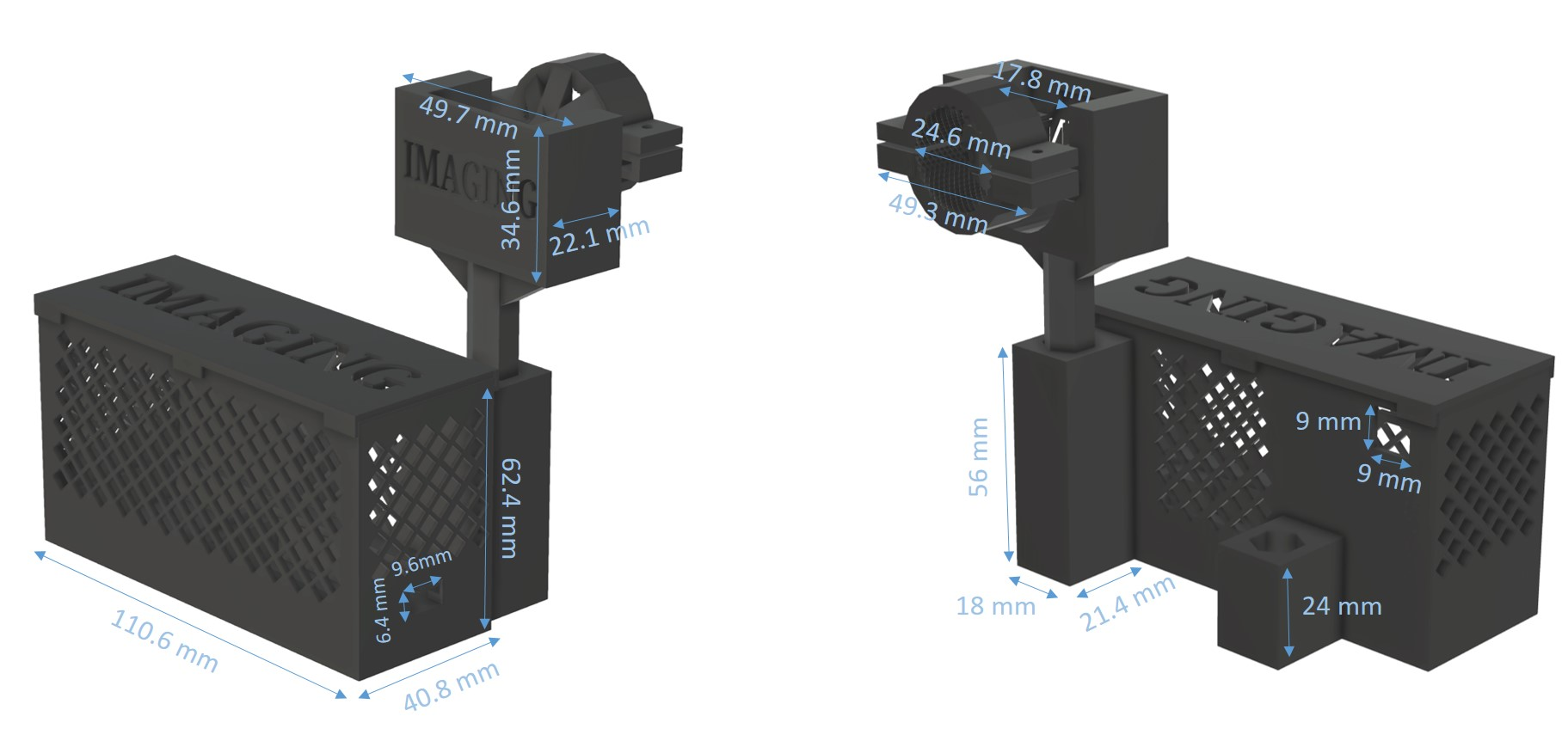

Supplement: S3 Fig — (TIFF) [file pone.0304085.s004.tiff]

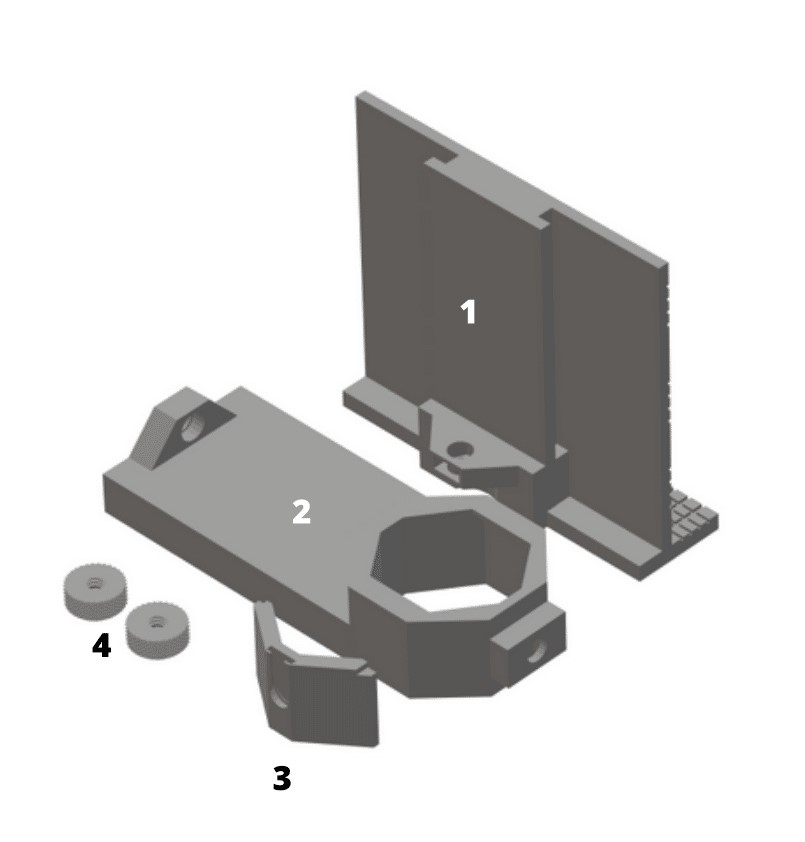

Supplement: S6 Fig — (1) Smartphone holder; (2) Adapter support; (3) Ocular lens hitch; (4) Wheels for regulating dimension of the screws. (TIFF) [file pone.0304085.s007.tiff]
